# Supplementary material for: FastqPuri: high-performance preprocessing of RNA-seq data
Source: BMC Bioinformatics. 2019 May 3;20:226. doi: 10.1186/s12859-019-2799-0 (PMC6500068; doi:10.1186/s12859-019-2799-0)
Supplement: Supplementary file 2 — Archive of FastqPuri. Archive containing all files needed to install and run FastqPuri v1.0.6. Date stamp March 22, 2019. (GZ 47,819 kb) [file 12859_2019_2799_MOESM2_ESM.gz › FastqPuri-1.0.6/html/str__manip_8h_source.html]

FastqPuri: include/str\_manip.h Source File


FastqPuri


- include

str\_manip.h

Go to the documentation of this file.

1 /\*\*\*\*\*\*\*\*\*\*\*\*\*\*\*\*\*\*\*\*\*\*\*\*\*\*\*\*\*\*\*\*\*\*\*\*\*\*\*\*\*\*\*\*\*\*\*\*\*\*\*\*\*\*\*\*\*\*\*\*\*\*\*\*\*\*\*\*\*\*\*\*\*\*\*\*

2  \* Copyright (C) 2017 by Paula Perez Rubio \*

3  \* \*

4  \* This file is part of FastqPuri. \*

5  \* \*

6  \* FastqPuri is free software: you can redistribute it and/or modify \*

7  \* it under the terms of the GNU General Public License as \*

8  \* published by the Free Software Foundation, either version 3 of the \*

9  \* License, or (at your option) any later version. \*

10  \* \*

11  \* FastqPuri is distributed in the hope that it will be useful, \*

12  \* but WITHOUT ANY WARRANTY; without even the implied warranty of \*

13  \* MERCHANTABILITY or FITNESS FOR A PARTICULAR PURPOSE. See the \*

14  \* GNU General Public License for more details. \*

15  \* \*

16  \* You should have received a copy of the GNU General Public License \*

17  \* along with FastqPuri. \*

18  \* If not, see <http://www.gnu.org/licenses/>. \*

19  \*\*\*\*\*\*\*\*\*\*\*\*\*\*\*\*\*\*\*\*\*\*\*\*\*\*\*\*\*\*\*\*\*\*\*\*\*\*\*\*\*\*\*\*\*\*\*\*\*\*\*\*\*\*\*\*\*\*\*\*\*\*\*\*\*\*\*\*\*\*\*\*\*\*\*\*/

20

29 #ifndef STR\_MANIP\_H\_

30 #define STR\_MANIP\_H\_

31

32

36 typedef struct \_split {

37  int N;

38  char \*\*s;

39 } Split;

40

41

42 int str\_isascii(char\* s);

43

44 int strindex(char \*s, char \*t);

45

46 int count\_char(char \*s, char c);

47

48 Split strsplit(char \*str, char sep);

49

50 #endif // endif STR\_MANIP\_H\_

count\_char

int count\_char(char \*s, char c)

returns the # of occurences of char c in string s

**Definition:** str\_manip.c:67

\_split

contains a splitted string and the number or splitted fields

**Definition:** str\_manip.h:36

Split

struct \_split Split

contains a splitted string and the number or splitted fields

\_split::s

char \*\* s

**Definition:** str\_manip.h:38

strsplit

Split strsplit(char \*str, char sep)

Separates strings by a separator.

**Definition:** str\_manip.c:94

strindex

int strindex(char \*s, char \*t)

returns index of t in s (start, first occurence)

**Definition:** str\_manip.c:53

\_split::N

int N

**Definition:** str\_manip.h:37

str\_isascii

int str\_isascii(char \*s)

return nonzero iff all elements in the string are in the ASCII set.

**Definition:** str\_manip.c:39


---

Generated on Mon Mar 19 2018 23:42:01 for FastqPuri by  

 1.8.14
